# Breast Cancer Radiotherapy Plan Metrics: A Principal Component Analysis Using the Mean Absolute Dose Deviation

**DOI:** 10.7759/cureus.117662

**Published:** 2026-10-08

**Authors:** Olena Gorobets, Meymey San, Linda Mrissa, Sopheary Yoeun, Zineb Dahbi, Minh Dung Nguyen, Steve Heymann, Nam P Nguyen, Jeffrey M Ryckman, Vincent Vinh-Hung, Parvez Shaikh

**Affiliations:** 1 Maxillofacial Surgery, Cancer Tech Care Association, Perpignan, FRA; 2 Radiotherapy, Khmer Soviet Friendship Hospital, Phnom Penh, KHM; 3 Radiotherapy, University Hospital of Martinique, Fort-de-France, FRA; 4 Radiotherapy, International University Hospital Cheikh Khalifa, Mohammed VI University of Health Sciences (UM6SS), Casablanca, MAR; 5 Orthopedics and Sport Medicine, Ho Chi Minh City Sports Medicine Association, Ho Chi Minh City, VNM; 6 Radiotherapy, Strasbourg Oncologie Libérale, Strasbourg, FRA; 7 Radiation Oncology, Howard University, Washington, D.C., USA; 8 Executive Committee, The International Geriatric Radiotherapy Group, Washington, D.C., USA; 9 Radiation Oncology, West Virginia University, Camden Clark Medical Center, Parkersburg, USA; 10 Radiotherapy, Centre Hospitalier Public du Cotentin, Cherbourg-en-Cotentin, FRA; 11 Executive Committee, The International Geriatric Radiotherapy, Washington, D.C., USA; 12 Radiotherapy, Universitair Ziekenhuis Brussel, Brussels, BEL; 13 Radiation Oncology, West Virginia University, Morgantown, USA

**Keywords:** dose volume histogram, factorial design, mean absolute dose deviation, principal component analysis, statistics, treatment planning

## Abstract

Introduction

Radiotherapy for breast cancer must deliver a sufficient dose to the targets while limiting the dose to the heart, lungs, and contralateral breast. Treatment plans are evaluated on many dose-volume metrics at once, and the balance between target coverage and organ sparing depends on the patient’s setup and on the radiotherapy technique. We investigate the relationships among doses to targets, organs, and patient and treatment factors, and examine whether principal component analysis (PCA) can display these relationships in a small factorial planning dataset.

Methods

PCA was applied to the data from a quasi-experimental two-level factorial treatment planning study of breast cancer adjuvant radiotherapy. The quasi-experiment assessed all combinations of five variables in eight patients: tumor laterality (left vs. right), surgery (mastectomy vs. breast-conserving), target volume (with vs. without lymph nodes), patient’s setup position (supine vs. prone) (each patient underwent simulation in the two positions), and radiation delivery (conventional radiotherapy vs. tomotherapy) (both plans were computed in each setup position), which resulted in 32 distinct combinations of the variables.

Results

PCA showed that tumor left laterality and prone setup were associated with higher heart and contralateral breast doses; nodal irradiation and conventional radiotherapy were associated with higher lung doses, whereas post-mastectomy radiation contributed to high doses to both heart and lung. Tomotherapy was associated with reduced lung doses, better target coverage, slightly reduced heart doses, and increased contralateral breast doses.

Conclusion

PCA on a factorial design identified correlations between treatment factors and dosimetric outcomes with a minimal number of patients, which might be important in exploratory research, reducing the initial burden of accruing patients.

## Introduction

Adjuvant radiotherapy reduces breast cancer recurrence and mortality, but the gain can be offset by late cardiac and pulmonary toxicity that is related to the doses received by the heart and lungs [1-3]. Treatment plan evaluation therefore weighs coverage of the targets against sparing of organs at risk across a large number of dose-volume metrics. Comparing plans across many patients and many metrics quickly becomes unmanageable by pairwise inspection.

Principal component analysis (PCA) is a classical multivariate technique that re-expresses a table of many correlated measurements as a small number of uncorrelated composite axes, ordered by the amount of variability they capture [4]. Its output, the biplot, displays in one picture which measurements vary together, which vary in opposite directions, and which cases drive the differences. PCA is well established in ecology and chemometrics but is seldom applied to radiotherapy plan metrics.

Radiotherapy treatment plan evaluation is at the core of the radiotherapy process. For any individual patient treatment, there are numerous techniques to deliver a given dose to the targets without incurring excess doses to the organs. The actual treatment applied to a patient is delivered according to a treatment plan considered best among several alternatives. Treatment plans are summarized by cumulative dose-volume histograms (DVHs) that display the statistics of the doses delivered to the organs and targets. The mean absolute dose deviation (MADD) is a measure of dispersion proposed as a DVH metric [5]. The MADD measures the area between the DVH of a structure and the oncologist's specified dose reference for that structure. In a perfect treatment plan, the doses delivered would exactly equate to the specified dose references, either zero for an organ (ideally an organ should receive no dose) or the prescribed dose for a target volume (ideally a target should receive the dose prescribed, not less, not more). That is, the hypothetical perfect plan would show no deviation from the dose references for any organ or target structure. The MADDs are expressed as physical radiation doses, in the same unit as the dose prescription, regardless of the type of structure [6]. That property and the same built-in concept of dispersion applied to all structures allow combining the MADDs of organs and targets into a physically coherent penalty score, detailed later, which summarizes how far a plan under evaluation deviates from the ideal plan delivering zero dose to organs and exactly the prescribed dose to targets [7].

This paper presents the use of the MADDs and the penalty score to obtain a view of dosimetric outcomes with the help of PCA. Herein, PCA was applied to a multifactorial study of adjuvant radiotherapy for breast cancer [6], in which five patient- and treatment-related factors were varied systematically. Five variables and levels that might impact the dosimetric outcomes were taken into consideration: the laterality of the tumor (left or right), the type of surgery (mastectomy or breast-conserving), the extent of clinical targets (regional nodal irradiation or not), the patient's setup position (prone or supine), and the radiation delivery technique (tomotherapy or conventional radiotherapy). We examine how PCA displayed the relationship of these factors with the dosimetric results and the penalty score and whether the display, on a deliberately small factorial dataset, is consistent with known relationships between treatment factors and dosimetric outcomes.

## Materials and methods

Source study

The source study was conducted in 2006-2007 at the Universitair Ziekenhuis Brussel as a pre-trial dosimetric evaluation before the TomoBreast randomized trial (NCT00459628) [6]. Conventional radiotherapy used tangential breast/chest wall fields and, where indicated, anterior axillary-supraclavicular fields, with additional multileaf collimator segments, planned on Pinnacle3 with the collapsed cone convolution algorithm. Tomotherapy plans were inverse-planned on the TomoTherapy Hi-Art treatment planning system (Accuray Inc., Sunnyvale, CA). All plans were prospectively approved within the department's clinical workflow. The original analysis of that study [2] used the penalty score as an outcome variable in a Lenth analysis of an unreplicated 2^5 factorial design; it identified nodal irradiation, left laterality, and mastectomy as penalizing factors, and a favorable interaction of tomotherapy with nodal irradiation.

The present study re-examines the same 32 plans with a different question: rather than testing which factors change the score, we ask how factors, dose-volume metrics, and the score co-vary. The study population was composed of eight adult patients presenting with a primary unilateral breast cancer referred for postoperative radiotherapy who were consecutively selected if they presented with a unique combination of surgery type, nodal involvement, and laterality that had not yet been included, providing 2x2x2=8 distinct patient-related factor levels. All selected patients underwent, after informed consent, a double simulation in supine and prone setup positions. Computed tomography images were acquired in each position. Structures delineated for planning included planning target volumes (PTV), PTVbw (target volume breast after breast-conserving surgery, or chest wall after mastectomy), PTVn (target volume regional nodes), and organs at risk (OAR): ipsilateral lung, heart, and contralateral breast (CB). Treatment plans were computed for tomotherapy and for conventional radiotherapy. Thus, each patient underwent two simulation setups, supine and prone, each of which received two plans, tomotherapy and conventional, providing 2x2=4 treatment-related factor levels, hence totaling 4x8=32 distinct combinations. The prone setup was applied to all patients, including those with mastectomy, because the original protocol treated setup position as a factor of the full factorial design. The dataset is closed: no additional patients can be accrued, the data are anonymized, and the analysis is descriptive. The dose prescription was 42 Gy in 15 fractions of 2.8 Gy daily, the institutional hypofractionation schedule of the TomoBreast trial, comparable in biologically effective dose to the Canadian 42.5 Gy/16 fractions and START B 40 Gy/15 fractions regimens [8-10].

The MADDs were computed for all structures across all treatment combinations. The penalty score pertaining to a planning combination was constructed using a weighted sum of the MADDs: $\text{Penalty Score} = \sum_{i=1}^{5} w_i \times M_i(A_i)$, where *Mi* is the MADD of the structure *i*, with i=1...5 assigned to PTVbw, PTVn, ipsilateral lung, heart, and contralateral breast, *Ai* is the reference dose, 42 Gy for PTVbw and PTVn, 0 Gy for organs, *wi* is the weight assigned to the structure *i*, with ∑wi = 1. The constraint ∑wi = 1 makes the penalty score a weighted mean of the MADDs, expressed in Gy on the same scale as the prescription; a different constant would only rescale the score. As in [6], the same weights were assigned to the structures, all = 0.20. The theoretical best possible penalty score is 0 when all MADDs are 0. High scores indicate poor dosimetric outcomes. The score has no practically useful upper bound: it is bounded only by the weighted mean of the maximum physically deliverable doses (for a hypothetical uniform 100 Gy exposure, 0.2 × (100+100+100+58+58) = 83.2 Gy). Three analyses were conducted: PCA with the equally weighted score, PCA without the score, and a sensitivity analysis over alternative weightings, described below.

Dosimetric data are dose-volume metrics computed from the treatment plans for each structure: Dmax, maximum dose to the structure; Dmin, minimum dose to the structure; M or MADD, mean absolute dose deviation to the structure; V5Gy, V17Gy, V20Gy, V95, V110, volume of the structure receiving 5 Gy, 17 Gy, 20 Gy, 95%, and 110% of the prescribed dose, respectively; and hotspots, the highest dose observed outside the PTVs. Lung V5Gy was not among the metrics recorded in 2007; it was obtained for the present revision by linear interpolation at 5 Gy on the cumulative dose-volume histogram of the ipsilateral lung of each plan, digitized for the source study [6]. As a check of the digitization, V17Gy and V20Gy, interpolated from the same curves, reproduced the values recorded in 2007 with a mean absolute difference of 0.14 percentage points (maximum 0.34). These data are continuous and will be referred to as “dosimetric measures”. They were evaluated for each distinct combination of breast laterality, surgery type, nodal irradiation, setup position, and radiotherapy technique, which we will refer to as qualitative variables, or factors identified by their levels (e.g., variable “laterality”, factor levels “left” or “right”). Thus, the data is a highly multidimensional table, in which we will organize the rows as nominal cases, each representing a distinct combination, and the columns as variables, representing the qualitative factors and the dosimetric measures. Note that the five factors are input variables, not principal components; the principal components are the derived axes returned by the analysis, each a weighted combination of all input variables.

To reduce the dimension, i.e., reduce the complexity to make the structure of the data clearer, PCA re-expresses that data by a linear transformation of the original variables into principal components, such that the first principal component explains the greatest variability of the data, the second principal component explains the next highest variability, and so on, and such that the principal components are uncorrelated [4]. Retaining a pair of principal components, the results can then be displayed on a biplot, where the cases (usually represented as dots) and the variables (usually represented as arrows) are plotted according to their coordinates expressed in terms of the principal components. The interpretation will be recalled later in the presentation of results. Briefly, a higher contribution of cases to variability would be represented by a farther distance from the origin of the principal components. Positively correlated variables would be represented by arrows pointing in the same direction, lack of correlation between a pair of variables would be represented by orthogonal arrows, and negative correlation would be indicated by arrows pointing in opposite directions.

The PCA was extended to tables with qualitative variables using the Hill and Smith analysis [11]. The Hill and Smith analysis handles a table mixing quantitative and qualitative columns. Quantitative variables are centered and scaled to unit variance; each qualitative variable is expanded into indicator columns for its levels, centered and scaled by level frequency, so that the two kinds of columns contribute comparably to the total inertia. The eigen-decomposition of the resulting weighted cross-product matrix gives the eigenvalues (the variance captured by each component) and the eigenvectors (the loadings of the variables); case scores are the projections of the rows on the eigenvectors. The sensitivity of the display to the weights of the penalty score was examined by recomputing the score and the PCA under nine weightings: equal weights; each structure in turn given 20-fold priority (PTVbw, PTVn, heart, lung, contralateral breast); both PTVs jointly prioritized; and PTV:OAR = 50:50 (0.25, 0.25, 1/6, 1/6, 1/6) and 60:40 (0.30, 0.30, 2/15, 2/15, 2/15). For each weighting, the angle between the score arrow and each factor level in the plane of the first two components was recorded. The statistical implementation used the R software version 4.3.2 (The R Foundation, Vienna, Austria) [12], applying the function dudi.hillsmith from the R package ade4 version 1.7-22 [11], and the package factoextra version 1.0.7 for additional plots. The data table, the R script, and all outputs are openly available [13].

## Results

Table 1 shows the dosimetric measures (columns of structure doses and hotspots) pertaining to each combination (identified by the 'factors' columns) and the corresponding penalty scores (last column). The rows identify the distinct combinations of factors.

**Table 1:** Dosimetric results. ID, patient number. Surg, surgery. Tum, tumorectomy. Mast, mastectomy. RT, radiotherapy. Tech, technique. TT, tomotherapy. CR, conventional radiotherapy. S, supine. P, prone. N0: no nodal radiation. N+: nodal irradiation. PTV: planning target volume. M: Mean absolute dose deviation. NA: not available; assigned 0 for the calculation of the penalty score. Hotspot: max dose as % prescription outside of PTV.

ID	Factors					PTV breast/chest wall			PTV lymph nodes			Lung				Heart				Contralateral Breast			Hot-spots	Score
	Side	Surg	Nodal RT	Tech	Setup	V95 (%)	V110 (%)	M (Gy)	V95 (%)	V110 (%)	M (Gy)	V5Gy (%)	V17Gy (%)	V20Gy (%)	M (Gy)	Dmax (Gy)	V17Gy (%)	V20Gy (%)	M (Gy)	Dmax (Gy)	Dmin (Gy)	M (Gy)	(%)	(Gy)
4	Right	Tum	N0	TT	S	99.80	0.00	0.80	0.00	0.00	NA	11.08	12.99	11.08	6.87	3.01	0.00	0.00	1.09	1.87	0.11	0.33	109.8	1.82
					P	98.60	0.00	0.82	0.00	0.00	NA	1.39	1.70	1.39	1.77	10.44	0.00	0.00	2.44	23.47	2.23	7.25	108.8	2.46
				CR	S	97.96	0.00	1.18	0.00	0.00	NA	8.70	9.20	8.70	4.52	1.62	0.00	0.00	0.33	0.43	0.15	0.01	109.2	1.21
					P	98.70	0.00	1.28	0.00	0.00	NA	3.80	4.45	3.80	2.67	40.92	5.80	5.40	3.40	38.81	0.01	0.50	111.4	1.57
2	Right	Tum	N+	TT	S	99.60	0.00	0.46	97.15	0.00	0.52	13.99	16.41	13.99	7.69	4.95	0.00	0.00	0.94	2.01	0.25	0.49	105.1	2.02
					P	92.40	0.00	1.43	86.25	0.00	2.11	7.92	9.08	7.92	4.67	3.97	0.00	0.00	1.01	4.87	0.34	0.87	104.8	2.02
				CR	S	95.70	0.00	1.08	36.95	0.00	8.10	18.80	20.50	18.80	8.74	3.46	0.00	0.00	0.53	0.67	0.00	0.09	107.2	3.71
					P	95.34	0.04	1.58	43.34	2.50	3.52	5.30	6.10	5.30	3.26	2.46	0.00	0.00	0.61	39.79	0.01	1.53	115.3	2.10
8	Right	Mast	N0	TT	S	99.87	0.00	0.83	0.00	0.00	NA	7.98	9.58	7.98	4.86	1.35	0.00	0.00	0.48	2.45	0.13	0.33	105.2	1.30
					P	99.79	0.00	1.12	0.00	0.00	NA	5.00	6.12	5.00	3.31	4.64	0.00	0.00	0.63	2.05	0.20	0.44	109.0	1.10
				CR	S	97.68	0.00	0.86	0.00	0.00	NA	6.00	6.74	6.00	3.43	0.80	0.00	0.00	0.14	0.19	0.01	0.06	109.7	0.90
					P	97.93	0.00	1.36	0.00	0.00	NA	5.00	6.27	5.00	3.61	2.92	0.00	0.00	0.23	5.40	0.01	0.11	110.4	1.06
5	Right	Mast	N+	TT	S	95.00	0.00	1.28	93.00	0.00	1.23	11.63	13.85	11.63	6.68	5.39	0.00	0.00	0.87	3.02	0.23	0.48	109.8	2.11
					P	97.30	0.00	0.93	84.54	0.40	2.22	9.80	11.17	9.80	6.01	4.33	0.00	0.00	1.00	16.75	0.40	1.01	113.3	2.23
				CR	S	98.86	0.25	1.25	85.12	0.00	2.57	21.37	23.43	21.37	10.0	2.46	0.00	0.00	0.44	9.41	0.01	0.17	111.1	2.89
					P	92.20	0.00	0.95	57.20	0.06	3.35	18.00	20.20	18.00	7.95	6.60	0.00	0.00	0.70	40.65	0.02	2.60	113.9	3.11
1	Left	Tum	N0	TT	S	96.76	0.00	0.71	0.00	0.00	NA	8.84	10.49	8.84	5.60	36.55	1.51	1.04	4.80	2.39	0.20	0.53	105.5	2.33
					P	97.66	0.00	0.56	0.00	0.00	NA	0.46	0.73	0.46	1.55	34.90	1.10	0.74	3.20	38.45	1.41	8.58	105.2	2.78
				CR	S	96.50	0.00	1.05	0.00	0.00	NA	6.50	7.30	6.50	4.02	41.00	1.77	1.50	1.88	1.01	0.60	0.11	105.6	1.41
					P	98.70	0.00	1.04	0.00	0.00	NA	3.80	4.45	3.80	1.67	40.92	5.80	5.40	3.40	38.81	0.01	0.50	110.8	1.32
7	Left	Tum	N+	TT	S	99.05	0.00	1.51	94.50	0.00	1.91	12.40	16.20	12.40	7.59	19.31	0.02	0.00	1.07	2.69	0.25	0.49	105.2	2.51
					P	98.33	0.00	0.81	90.10	0.00	1.92	9.40	12.00	9.40	5.58	36.64	0.65	0.44	1.57	5.13	0.45	0.80	106.2	2.13
				CR	S	98.40	0.01	1.25	67.50	0.00	2.78	21.20	22.80	21.20	9.43	37.60	0.25	0.20	0.74	0.35	0.01	0.11	110.3	2.86
					P	97.10	0.00	0.90	81.60	0.00	2.54	10.40	11.20	10.40	5.09	38.56	0.60	0.45	0.89	4.49	0.01	0.25	110.1	1.93
6	Left	Mast	N0	TT	S	96.50	0.00	1.10	0.00	0.00	NA	7.48	9.20	7.48	4.70	23.44	0.08	0.03	1.58	18.76	0.17	3.11	106.2	2.10
					P	95.70	0.00	1.50	0.00	0.00	NA	8.72	11.40	8.72	9.30	39.01	8.00	4.34	6.17	25.88	0.53	3.85	108.3	4.16
				CR	S	96.06	0.00	1.07	0.00	0.00	NA	7.10	7.80	7.10	3.49	41.37	1.15	0.95	0.95	0.73	0.01	0.07	107.9	1.12
					P	92.70	0.40	1.80	0.00	0.00	NA	13.10	14.30	13.10	6.29	43.20	4.60	4.10	2.53	47.01	0.03	6.25	112.0	3.37
3	Left	Mast	N+	TT	S	98.56	0.00	0.82	76.24	0.00	2.58	14.00	16.10	14.00	6.99	38.99	4.60	3.24	3.37	17.35	0.40	3.56	110.2	3.46
					P	98.68	0.00	0.78	33.56	0.00	9.10	6.68	7.45	6.68	3.99	38.78	13.95	12.69	6.60	31.07	1.15	11.2	108.4	6.34
				CR	S	98.43	0.00	1.49	62.60	0.00	3.90	19.20	21.20	19.20	7.45	40.46	5.20	4.50	3.20	6.82	0.01	0.02	110.0	3.21
					P	98.37	0.00	1.31	57.80	0.00	5.15	15.00	16.05	15.00	7.47	43.15	30.90	29.90	13.3	31.57	0.01	0.51	113.7	5.55

The PCA was applied to the table. The first two principal components represented 45.1% of the variability of the data; the third component contributed 12.8% and the remaining components decreasingly to less than 0.1% of the total variation (Table 2).

**Table 2:** Eigenvalues, proportion, and cumulative proportion of inertia of the principal components (analysis with equal weights and lung V5Gy).

Component	Eigenvalue	Percent	Cumulative
1	5.83	24.30	24.30
2	4.99	20.80	45.10
3	2.96	12.30	57.40
4	2.26	9.40	66.80
5	1.76	7.30	74.20
6	1.37	5.70	79.90
7	0.94	3.90	83.80
8	0.73	3.10	86.90
9	0.70	2.90	89.80
10	0.65	2.70	92.50
11	0.55	2.30	94.80
12	0.41	1.70	96.50
13	0.30	1.20	97.70
14	0.20	0.80	98.60
15	0.14	0.60	99.10
16	0.07	0.30	99.50
17	0.07	0.30	99.70
18	0.04	0.10	99.90
19	0.02	0.10	100.00
20	0.01	0.00	100.00
21	0.00	0.00	100.00
22	0.00	0.00	100.00
23	0.00	0.00	100.00
24	0.00	0.00	100.00

The coordinates of the 32 plans on the first two components are listed in Table 3.

**Table 3:** Coordinates of the 32 plans on the first two principal components, with patient, factor levels, and equal weights penalty score.

Case number	Case label	Patient	Axis1	Axis2
1	c4TS	4	-1.391	-1.791
2	c4TP	4	-4.082	2.921
3	c4CS	4	-2.167	-1.569
4	c4CP	4	-1.814	2.090
5	c2TS	2	0.068	-2.811
6	c2TP	2	-0.709	-1.098
7	c2CS	2	2.526	-2.847
8	c2CP	2	-0.481	0.332
9	c8TS	8	-2.329	-1.493
10	c8TP	8	-2.814	-0.302
11	c8CS	8	-2.535	-1.312
12	c8CP	8	-2.194	-0.574
13	c5TS	5	0.685	-2.253
14	c5TP	5	0.247	-0.793
15	c5CS	5	3.337	-3.375
16	c5CP	5	2.506	-1.020
17	c1TS	1	-1.155	0.137
18	c1TP	1	-3.631	4.044
19	c1CS	1	-2.004	0.180
20	c1CP	1	-1.863	2.497
21	c7TS	7	1.130	-2.013
22	c7TP	7	-0.046	-0.313
23	c7CS	7	2.899	-2.493
24	c7CP	7	0.319	-0.514
25	c6TS	6	-1.442	0.281
26	c6TP	6	2.217	1.967
27	c6CS	6	-1.651	-0.182
28	c6CP	6	2.058	2.198
29	c3TS	3	2.002	0.294
30	c3TP	3	2.276	5.638
31	c3CS	3	3.449	-0.968
32	c3CP	3	6.589	5.142

Figure 1's main area plots the PCA-derived penalty score (orange arrow), the qualitative variable levels (red arrows), and the dosimetric measures (black arrows), using as coordinates their first two principal component scaled scores. The vectors indicate how much the factors and the dosimetric measures correlate. Two arrows that are perpendicular indicate no correlation between the factors they represent; if the angle is less than 90°, they are positively correlated; the smaller the angle, the stronger the correlation; if the angle is more than 90°, they are negatively correlated. The factor levels are binarily opposed, hence perfectly negatively correlated: the laterality left arrow is the same length as the laterality right arrow, but opposite in direction, likewise with the other variables of nodal irradiation, surgery, setup, and radiation delivery. The distance from the origin indicates the relative contribution of a variable to the variability of the data.

**Figure 1:**
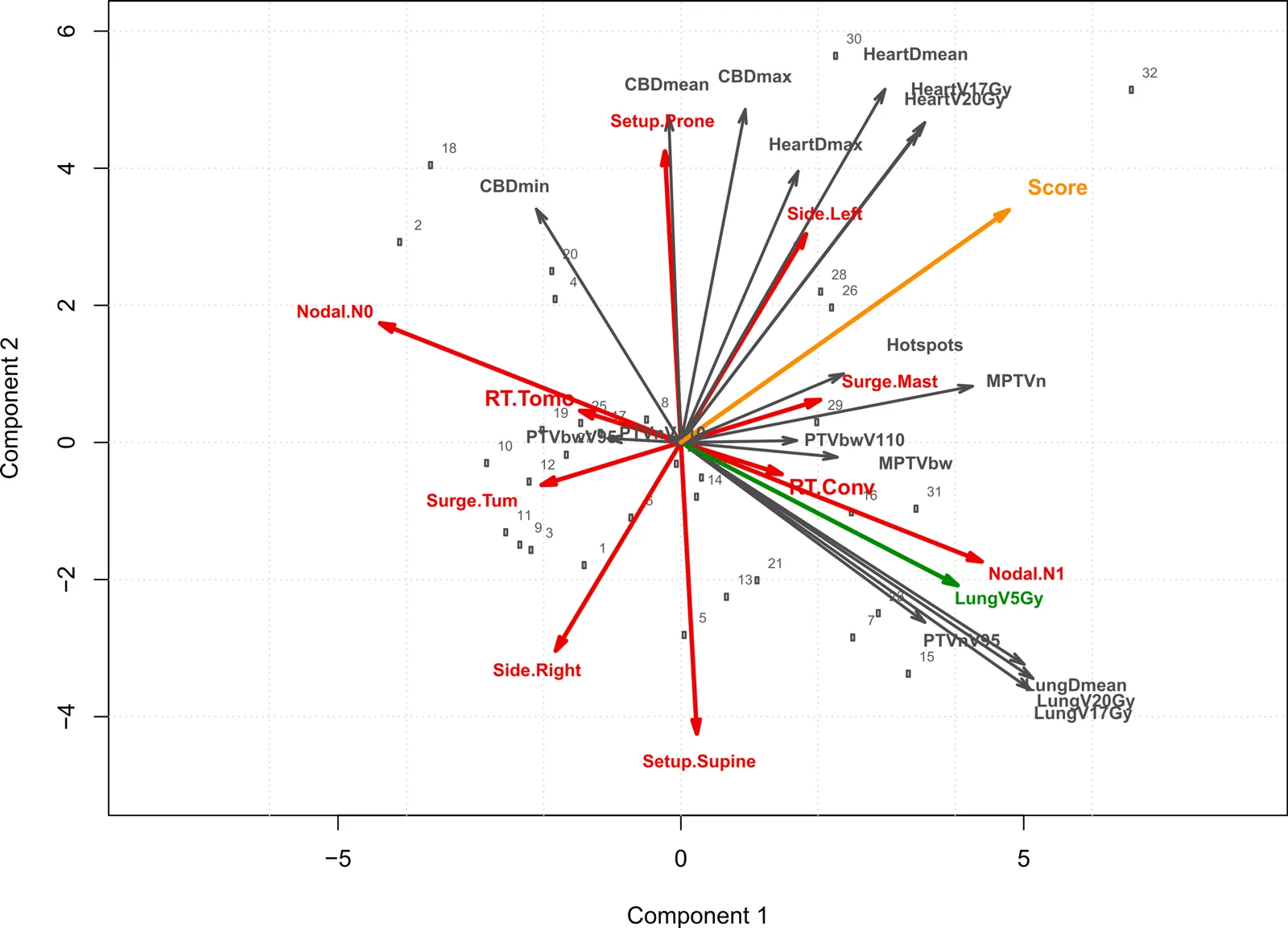
Principal component analysis of Table 1, with lung V5Gy. Arrows identify the factors (red), the dosimetric variables (black; lung V5Gy in green), and the penalty score (orange). Dots identify the individual rows, numbered as in Table 3. Coordinates are proportional to the correlations with the first two principal components.

Overall, Figure 1 displays two distinct groups of factors and dosimetric measures, on the left and the right side. Factors correlated with the penalty score (orange) appear on the right side, all at <90° of the score vector: prone, left breast, mastectomy, conventional irradiation, and N+ (nodal irradiation), clustered with higher doses to the contralateral breast, the heart, poor coverage of targets, and higher doses to the lung. There are strong correlations between the dosimetric measures of the heart (HeartDmean) and the contralateral breast (CBDmean). They project in the direction of the penalty score (orange arrow) on Figure 1’s upper right. Factors negatively correlated to the penalty score (opposite to penalization) appear on the left side.

Viewed part by part, on the graph’s upper-right, high heart doses relate with the left breast (left). On the graph’s lower-right, measures of lung doses (LungDmean, V20Gy, V17Gy, V5Gy) relate most closely with measures of high nodal dose (PTVnV95), with regional nodal irradiation (N1), and with conventional radiotherapy (RT.Conv). Lung V5Gy was strongly correlated with the lung mean dose (r = 0.87) and lay at 59° from the score arrow. Its mean was slightly higher with tomotherapy than with conventional radiotherapy (24.6% vs. 22.2%); one prone tomotherapy plan (patient 6, left mastectomy) had a V5Gy of 75.7%, the largest low-dose volume in the series. On the graph’s middle-right, poor coverage to the breast/chest wall and lymph nodes (indicated by the MADDs MPTVbw and MPTVn, by PTVbwV110, and Hotspots) are clustered with mastectomy (Surge.Mast). On the graph’s mid-upper part, high contralateral breast doses correlate with prone setup (Setup.Prone).

Because the original study was interested in the effect of radiotherapy technique, Figure 1 can be viewed with a focus onto the Tomotherapy arrow (RT.Tomo). Tomotherapy is opposite, i.e., negatively correlated, to the cluster of lung doses and nodal irradiation (lung, N+), and opposite to the cluster of poor target coverage (PTVn, PTVbw, Mastectomy). That is, tomotherapy reduced lung doses and improved target coverage. The heart dose arrows (heart cluster) are at >90° from the tomotherapy arrow, indicating improved heart doses with tomotherapy, though not markedly. The contralateral breast doses (CB cluster) are at <90° from the tomotherapy arrow, indicating a slightly increased contralateral breast dose with tomotherapy. Overall, tomotherapy improved on the treatment plan, as indicated by the large >90° angle between the score and the tomotherapy arrows.

A recurring question arose during revisions of this report: what would happen if other weights were assigned to the penalty score? Because the penalty score is a linear combination of the MADDs, changing the weights is expected to move the score arrow toward whichever structure receives the highest weight, leaving the relationships between the dose-volume metrics and the patient-therapy factors unchanged. This was verified by the sensitivity analysis (Figure 2, Table 4). Under all nine weightings, the dose-volume metrics and the factor levels kept the same relative positions, and the first two components captured 44.0-45.5% of the inertia. Only the score arrow rotated toward the prioritized structure: with priority to the heart, the score aligned with the heart metrics and left laterality (1-2°); with priority to the lung, with the lung metrics and nodal irradiation (1-6°); with priority to the contralateral breast, with the prone setup (2°). The PTV:OAR 50:50 and 60:40 weightings moved the score arrow by at most 12° relative to equal weights. Some panels of Figure 2 appear mirrored; this is the sign indeterminacy of principal components and does not alter the correlations.

**Figure 2:**
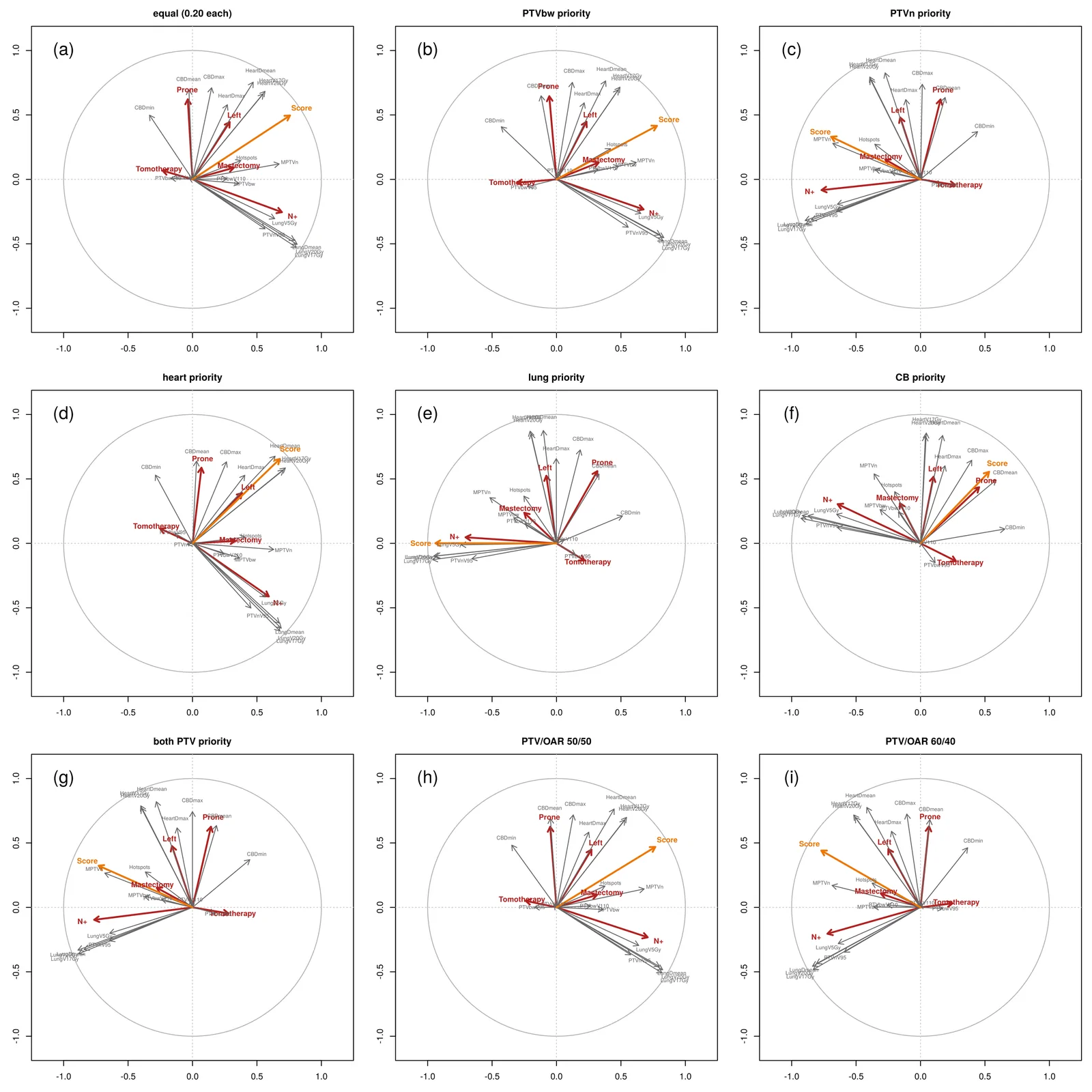
Sensitivity of the principal component display to the weights of the penalty score. Nine panels, one per weighting of the penalty score: (a) equal weights, 0.20 to each of the five structures; (b) priority to the breast/chest wall target; (c) priority to the nodal target; (d) priority to the heart; (e) priority to the lung; (f) priority to the contralateral breast; (g) priority to both targets equally; (h) 50% to the two targets and 50% to the three organs; (i) 60% to the two targets and 40% to the three organs. Each panel shows the variables in the plane of the first two principal components: factor levels (red), penalty score (orange), and dose-volume metrics (grey). Opposite factor levels are omitted. Panels may appear mirrored relative to one another owing to the sign indeterminacy of principal components; angles between arrows are unaffected. The figure was generated using R software version 4.3.2 (The R Foundation, Vienna, Austria) and the package ade4, using the script deposited in reference [13].

**Table 4:** Angle (degrees) between the penalty score arrow and each factor level, and the MADD of each organ, in the plane of the first two principal components, for nine weightings of the penalty score; last column, proportion of inertia captured by the first two components. Surge, surgery. Mast, mastectomy. RT, radiotherapy. Tomo, tomotherapy. N1: nodal radiation. CB, contralateral breast. PTV: planning target volume; bw, breast/chest wall; n, nodes. OAR, organ at risk. PC12, the proportion of inertia captured by the first two components.

Weighting	RT. Tomo	Setup. Prone	Side. Left	Surge. Mast	Nodal. N1	Lung V5Gy	Heart Dmean	Lung Dmean	CB Dmean	PC12
Equal	131	60	24	17	53	59	25	64	59	45.1
PTVbw	157	67	34	6	47	50	35	56	72	45
PTVn	164	79	46	4	32	42	46	46	81	44.1
Heart	111	39	1	41	79	80	2	87	43	45.4
Lung	149	119	81	43	4	1	83	6	121	45.5
CB	73	2	33	71	108	115	32	120	6	44
BothPTV	166	79	47	5	31	41	47	45	82	44.3
PTV_OAR 50_50	137	63	27	14	50	56	28	61	62	45
PTV_OAR 60_40	143	66	31	11	46	54	32	58	66	44.9

## Discussion

Correlations of the radiation dose metrics to organs and targets have received increasing attention [14]. The issue is complicated by the large number of possible combinations, multiplied by the choice of the types of metrics, e.g., mean dose, V20 Gy, and by the choice of the “granularity” of the measures, e.g., choosing a Vxx Gy every xx of 1 Gy, or 10 Gy, or else [15,16]. An exhaustive exploration of all pairwise combinations is practically unmanageable. An ongoing search for the best metric in breast radiotherapy in a single type of prescription technique already yielded 1.5 million pairwise comparisons [17], imposing either to reduce the number of structures to compare or to use a single metric, e.g., focusing on the mean only [14]. PCA is an alternative approach that can display in a snapshot the relationships among many variables and many measurements. The major contribution of the study is a summary well in line with common knowledge of how organs are affected in the radiotherapy of breast cancer: increased heart radiation exposure with left breast cancer [18], increased doses to the lung with nodal irradiation [19], and increased doses to the contralateral breast with prone setup [7]. Regarding tomotherapy, how it relates with the heart, contralateral breast, and lung doses is concordant with other studies of intensity-modulated techniques [20,21]. The behavior of lung V5Gy, added at revision, is likewise consistent with the known trade-off of rotational intensity-modulated techniques: lower V20Gy and mean lung dose at the price of a wider low-dose volume [20]. Of particular significance, the conditionally favorable effect of tomotherapy on lung and heart uncovered in this study has been confirmed in a prospective randomized clinical trial [22], 15 years after the present data.

PCA does not require the data to be organized according to a factorial design. However, that design provides several advantages that make it worth consideration, among which is the efficiency in identifying main effects and interactions [23]. Digressing back to the original factorial study [6], the original analysis was conducted using the penalty score as an outcome dependent variable, i.e., the penalty score was used as a measure of the effect on which patient-therapy factors acted [6]. Figure 3, resulting from that previous analysis, shows that among supine treatments, nodal irradiation was the foremost factor deteriorating plan quality, significantly increasing the penalty score by 1.33 Gy, p=0.001 (Figure 3, “N”, first horizontal bar). Tomotherapy done with nodal irradiation reduced the penalty score by 0.69 Gy, i.e., half the effect of nodal irradiation, p=0.026 (Figure 3, “TN”, second bar) [6]. The present PCA study differs in that we used the penalty score as a constructed factor alongside the other factors, without assigning it any specific role.

**Figure 3:**
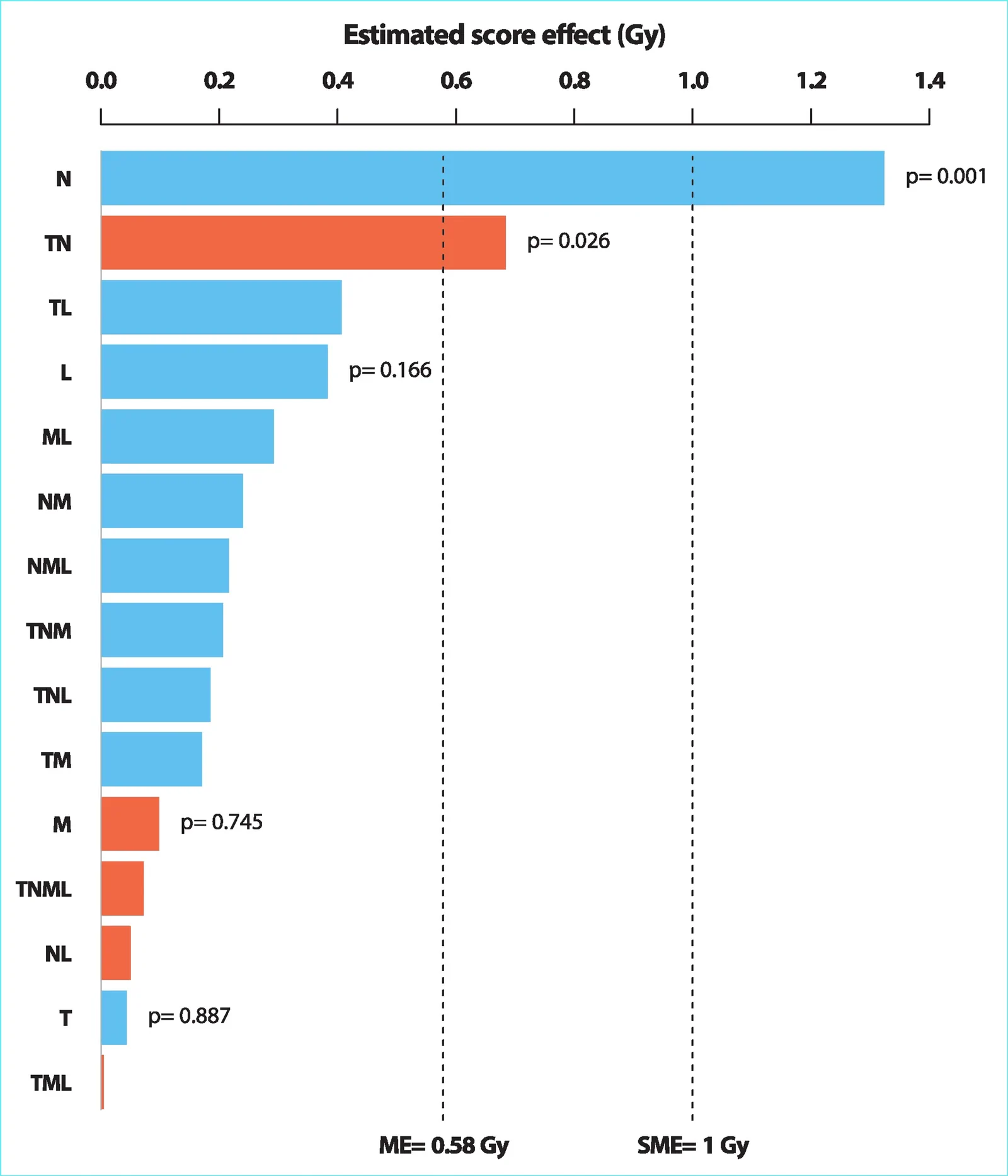
Factorial analysis of patient-therapy factors. N, nodal irradiation; T, tomotherapy; L, left breast; M, mastectomy; ME, margin of error of the factorial effects on the penalty score; SME, simultaneous margin of error. Blue, penalty increased by the factor or combination of factors; orange, penalty decreased. Reproduced from the authors’ own work [6] under a Creative Commons Non-Commercial CC BY-NC 4.0 license.

Limitations include the difficulty to reproduce the experimental conditions of this study: positioning mastectomy patients prone for the radiotherapy of regional nodes is not standard practice. Nothing in the present analysis argues for it: the prone setup clustered with mastectomy and left laterality on the penalized side of the biplot, consistent with the significant prone × mastectomy interaction found in the source study [6]. A separate consideration, not tested here, is that a prone setup reduces respiratory motion, a reason for which breast MRI is acquired prone, which may deserve evaluation for chest-wall irradiation with motion-sensitive techniques such as proton therapy. Since the first installations of tomotherapy in Europe, many other radiotherapy techniques have been developed. The expertise of dosimetrists and the efficiency of planning systems have considerably improved; hotspots seldom occur. The choice of the linear analyses was based on the interest in correlations between doses planned but cannot make assumptions on non-linear biological dose response. The source study noted that its runs were not randomized, were nested within patients, and were unreplicated [6]. These limitations carry over unchanged; the present analysis is descriptive and makes no inferential claim. Its added value is the visual integration of all metrics and factors in one display, and the finding that the display is robust to the weighting of the penalty score. Another limitation is the choice of weights for the penalty score. The penalty weights reflect the clinical importance attributed to doses to various structures, which is a clinical decision depending on the priority importance given to the heart, lungs, breast, or risk of secondary tumors. Treatment plans conducted without knowledge of the penalty naturally would be unaffected by whatever weights are chosen. Nevertheless, if a penalty score is used, the weights could affect the choice of a treatment plan [7]; the sensitivity analysis shows that the weights affect where the score points, not the relationships among the other variables.

Despite the limitations, a strength of the study derives from its factorial design. The design is omitted from medical statistics textbooks [24,25], but open references are readily accessible [23], including a defense tutorial that discusses issues of power analysis [26]. Conducting a conventional study using ordinary multiple regression to explore the dosimetric impact of five variables would require 90 to 184 patients [27,28]. Currently, the equivalent of nominally 32 patients was included, which was actually 8, because each patient underwent two dual interventions. That nominal number was sufficient to provide subgroup information all at once, with 2.8 to 5.75 times fewer patients than would have been needed in a conventional study. We agree that a larger sample would be preferable for confirmatory purposes; however, the dataset is closed, and the present results are descriptive and hypothesis-generating. Parsimonious sparing of human-clinical resources can be of critical importance. Accruing older and frail patients, usually excluded from clinical trials [29], is challenging, which adds to the hurdle of running costs usually beyond reach of many populations [30]. In such circumstances, efficient design minimizing study size is a necessity. We believe that the factorial setup might be encouraged in pilot studies to enhance the information yield. Combined with the MADD-based penalty score and PCA, the factorial design is a tool that could be increasingly valuable in future radiation therapy research.

## Conclusions

PCA of the dosimetric parameters and the MADD penalty score provides a directly interpretable overview of the dosimetric outcomes, facilitating the selection of radiation treatment plans that may require more intensive refinement. The overview was consistent with established clinical knowledge and was robust to the weighting of the penalty score. The factorial design on which it was conducted can help to optimize the information yield in prospective studies. The design and PCA on appropriate data can be considered for sparing human clinical resources.
